# Digital health as an enabler for hospital@home: A rising trend or just a vision?

**DOI:** 10.3389/fpubh.2023.1137798

**Published:** 2023-02-17

**Authors:** Kerstin Denecke, Richard May, Elizabeth M. Borycki, Andre W. Kushniruk

**Affiliations:** ^1^Bern University of Applied Sciences, Bern, Switzerland; ^2^Harz University of Applied Sciences, Wernigerode, Germany; ^3^School of Health Information Science, University of Victoria, Victoria, BC, Canada

**Keywords:** patient at home, hospital at home, SWOT, digital health, care model, innovation

## Abstract

**Background:**

Hospital@home is a model of healthcare, where healthcare professionals actively treat patients in their homes for conditions that may otherwise require hospitalization. Similar models of care have been implemented in jurisdictions around the world over the past few years. However, there are new developments in health informatics including digital health and participatory health informatics that may have an impact on hospital@home approaches.

**Objectives:**

This study aims to identify the current state of implementation of emerging concepts into the hospital@home research and models of care; to identify strengths and weaknesses, opportunities, and threats associated with the models of care; and to suggest a research agenda.

**Methods:**

We employed two research methodologies, namely, a literature review and a SWOT (strengths, weaknesses, opportunities, and threats) analysis. The literature from the last 10 years was collected from PubMed using the search string “*hospital at home*” *OR* “*care at home*” *OR* “*patient at home*.” Relevant information was extracted from the included articles.

**Results:**

Title and abstract review were conducted on 1,371 articles. The full-text review was conducted on 82 articles. Data were extracted from 42 articles that met our review criteria. Most of the studies originated from the United States and Spain. Several medical conditions were considered. The use of digital tools and technologies was rarely reported. In particular, innovative approaches such as wearables or sensor technologies were rarely used. The current landscape of hospital@home models of care simply delivers hospital care in the patient's home. Tools or approaches from taking a participatory health informatics design approach involving a range of stakeholders (such as patients and their caregivers) were not reported in the literature reviewed. In addition, emerging technologies supporting mobile health applications, wearable technologies, and remote monitoring were rarely discussed.

**Conclusion:**

There are multiple benefits and opportunities associated with hospital@home implementations. There are also threats and weaknesses associated with the use of this model of care. Some weaknesses could be addressed by using digital health and wearable technologies to support patient monitoring and treatment at home. Employing a participatory health informatics approach to design and implementation could help to ensure the acceptance of such care models.

## 1. Introduction

The aging of the population in Western countries, the increase in the number of citizens diagnosed with chronic diseases including a disproportionate rise in the cost of healthcare services, and an increase in the number of hospitals running out of resources place new demands on our national healthcare systems (1). These developments make it necessary to conceptualize, develop, implement, and innovate new solutions to provide patient care. One model of care that has already proven successful in some countries is hospital@home (2). Hospital@home refers to “a healthcare modality that provides active treatment by healthcare professionals in the patient's home for a condition that would otherwise require hospitalization” (3). Such programs typically involve multidisciplinary care teams delivering a bundle of services after early discharge or after an emergency room visit without the patient being hospitalized. The services include, among other things, home infusion, remote monitoring, and laboratory testing as well as home visits by nurses, physicians, nursing practitioners, and other related personnel such as social workers, physiotherapists, and pharmacists. Hospital@home treatment has proven to be particularly suitable for people with pneumonia, chronic obstructive pulmonary disease, or heart disease (4).

The World Health Organization (WHO) has recommended a series of strategies focusing on a person-centered approach aimed at providing and maintaining universal, equitable, high quality, and financially sustainable healthcare in future. One of the five strategic WHO goals refers to reorienting the model of care while another one addresses coordinating healthcare services (5). The concept of hospital@home falls directly within these strategic goals. Since most hospital@home care models have been established in some countries (for more than 15 years), the question arises whether developments over the last few years (including digitalization, participatory medicine, and participatory health informatics) have influenced the advancement of hospital@home as a model of care (6). Including information and communication technologies as part of hospital@home into care processes demonstrates the fundamental role of supporting a person-centered or participatory health approach (6). Participatory health informatics is a multidisciplinary field that applies information technology to medical conditions and analyses how the use of digital tools affects patients. It emphasizes individual-centered care, self-management, and decision-making while providing resources and delivering tools that enable active involvement (6). It is relevant for hospital@home because it allows for active engagement and collaboration between patients, healthcare providers, and researchers in the design, development, and use of technology for hospital@home programs. Such involvement helps ensure that the technology is responsive to the needs and preferences of patients and other stakeholders and improves the quality and effectiveness of healthcare. This can be especially important for hospital@home programs, as they are designed to provide care to patients in their homes, and patients may have different needs and preferences than they would have in a traditional hospital setting.

Overall, the objective of this study is to identify the current state of integration of these emerging concepts into hospital@home care. In particular, we are focused on the following research questions:

- Which stakeholders are involved in hospital@home concepts and realizations?
- Which medical conditions are considered for home treatment?
- Which services are provided at home?
- Which digital tools and technologies support hospital@home?
- What strategies are realized to achieve patient participation?

From the results of the review, we will derive strengths, weaknesses, opportunities, and threats of the hospital@home care model. We will also identify gaps in research in the context of WHO strategic goals as well as emerging trends in healthcare and health informatics such as participatory health informatics.

Existing reviews of hospital@home care approaches focus on the effectiveness and costs (2), factors associated with the workload of healthcare professionals (7), or with perceptions of patients and healthcare professionals (8). A Cochrane review published in 2016 found that “admission avoidance hospital at home, with the option of transfer to hospital, may provide an effective alternative to inpatient care for a selected group of elderly patients requiring hospital admission” (2). Caplan et al. (9) concluded in their meta-analysis that hospital@home approaches can lead to reductions in mortality, readmission rates, and cost and that the approaches have the potential to increase patient and caregiver satisfaction. In addition, patients tend to be more physically active at home. Therefore, patients are able to perform or improve their ability to perform activities of daily living for themselves more quickly (10). In the long term, hospital@home treatment is expected to be less costly than standard inpatient treatment in a hospital (2). Leff et al. (11) described a research agenda for hospital@home that was developed based on a survey distributed among conference participants (researcher, hospital@home physicians, and hospital@home program leaders). Ouchi et al. reported on the opportunities that arise from a hospital@home approach. However, their results are not based on a literature review (12). To the best of our knowledge, a review that studies the use of digital technologies in hospital@home and that identifies strengths, weaknesses, opportunities, and threats of hospital@home has yet to be published.

## 2. Methods

To achieve our research objectives, we employed two research methodologies, namely a literature review and a SWOT analysis (strengths, weaknesses, opportunities, and threats). This way, we are able to describe the landscape of the hospital@home literature and give practical implications regarding strengths, weaknesses, opportunities, and threats for both researchers and practitioners.

### 2.1. Literature review

**Figure 1:**
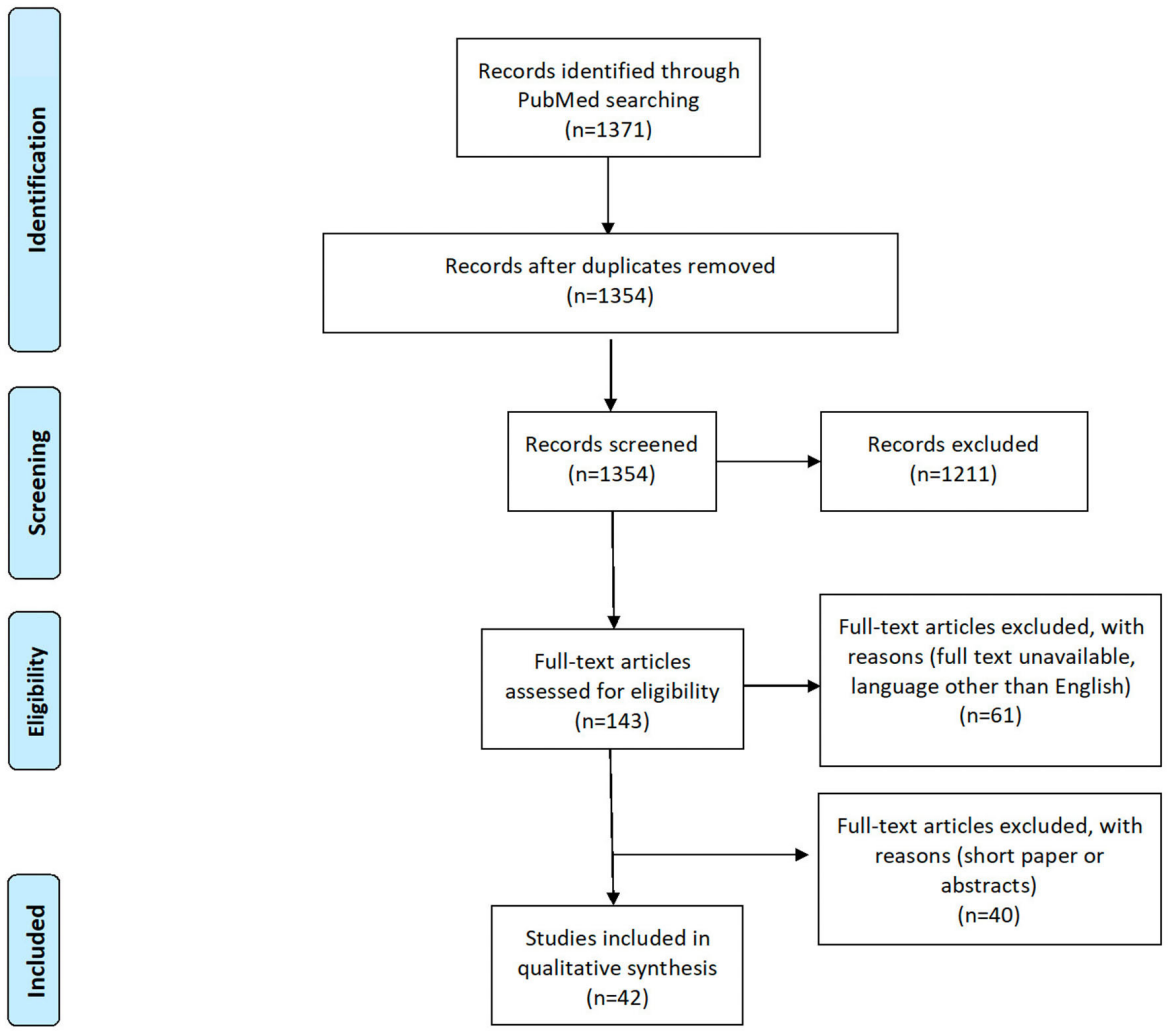
PRISMA flowchart.

We conducted a literature review based on the preferred items for systematic review and meta-analysis (PRISMA) statements (13), as summarized in Figure 1. Since our interest was in concrete concepts and implementations of hospital@home care instead of single enabling technologies for hospital@home, we intentionally resisted collecting data from technical databases like IEEE Xplore or the ACM Digital Library. Instead, we searched only in PubMed as this database lists articles from the healthcare domain without focusing only on technical aspects. To find appropriate literature, we created the following search string, consisting of hospital@home and its most prominent synonyms: “*hospital at home*” *OR* “*care at home*” *OR* “*patient at home*.”

Moreover, we defined the following five criteria to ensure the eligibility of our study:

1. The publication is written in English.
2. The publication is a peer-reviewed conference paper or journal article (see below).
3. The publication has been published between 2013 and 2022 (last decade).
4. The publication has at least five pages.
5. The publication presents concepts, architectures, theoretical frameworks, or concrete implementations related to hospital@home.

We intentionally focused on the last decade of research to cover the most recent research (criteria 3). We argue that developments around participatory health informatics, as well as digital health, mHealth, and digital health technology, might have entered the development of hospital@home concepts in the last few years. Criteria 4 was used to ensure a certain quality of the articles, assuming that a publication with a minimum number of pages provides enough details to comprehend the addressed problem. Posters, study protocols, and complete conference proceedings were excluded as well as articles dealing with end-of-life care, long-term care, midwifery, and nursing homes. The latter care settings are rather specific. In contrast, we are interested, in general, in hospital care delivered at home. We also excluded articles solely dealing with the perceptions of patients and healthcare professionals due to the focus on digitalization and technology involvement.

To extract relevant data from the retrieved literature, we defined the following 9 criteria in addition to typical bibliographic properties such as the year of publication:

- Country, where the approach was implemented.
- Practical orientation (realization and concept).
- Legal regulations mentioned (yes and no).
- Setting (early discharge, treatment at home, and other).
- Implemented care process/workflow.
- Involved stakeholders.
- Medical condition.
- Strengths, weaknesses, opportunities, and threats.
- Technologies involved.

We conducted the literature search on 6 July 2022. Overall, we identified 1,371 results on PubMed, after removing 1,354 duplicate results. Two authors (KD and RM) manually reviewed the articles by using the collaborative review tool Rayyan QCRI, which automatically removed 17 duplicates. Each reviewer examined half of the publications' titles and abstracts resulting in the inclusion of 143 articles. Next, the full texts were downloaded for detailed analyses in an Excel spreadsheet. However, for 16 articles, the full texts were not accessible, 13 articles had a wrong publication year (2012), and 32 were either in another language than English or out of topic, i.e., we considered 82 articles in the full-text review. All disagreements between the annotators were resolved in discussions until a consensus on a decision was achieved. During the full-text review, we excluded additional articles due to unmet inclusion criteria (number of pages: at least five pages), review articles, or when the article was not describing a concrete concept of hospital@home. Data were finally extracted from 42 articles (see Figure 1).

### 2.2. SWOT analysis

SWOT analysis is a method for identifying strengths, weaknesses, opportunities, and threats. The idea of a SWOT analysis originates in strategic management research (14), thus providing a highly practical orientation. Practical orientation refers to a focus or emphasis on applying knowledge, skills, and strategies in real-world situations or contexts. Adapting this to hospital@home research, we consider strengths and weaknesses as features of the hospital@home concepts themselves, or “internal” features. Conversely, opportunities include the economic, technical, social, political, legal, and environmental features representing the context of hospital@home. We thus consider opportunities to be “external” features. Threats are, similarly, external features that may prevent further real-world implementation of hospital@home concepts. From the retrieved articles, we collected and interpreted the results in terms of strengths, weaknesses, opportunities, and threats of hospital@home in general. Relevant questions driving our SWOT analysis are listed in Table 1.

**Table 1:** Questions driving the SWOT analysis.

**Internal features**	**Strengths**	**Weaknesses**
	• What is unique about hospital@home care models and related concepts? • What are advantages of hospital@home? • What are the greatest achievements of hospital@home care? • Could hospital@home care models already be used to significantly support the healthcare system in a public health crisis?	• What are disadvantages of hospital@home care models? • Is the concept sufficiently developed for the modern healthcare market? • Is hospital@home care useful for patients and is it accepted at all? • What needs improvement in the context of hospital@home?
**External features**	**Opportunities**	**Threats**
	• Which external changes in the context of healthcare concepts and the overall market will bring opportunities? • What are current trends supporting hospital@home care? • Is there any gap in the market that can be addressed by hospital@home? • Can hospital@home benefit from particular (health) technologies?	• What are current trends preventing hospital@home? • Are there serious concerns from patients that impair or prevent the actual implementation of concepts? • Is there enough actual motivation by the care providers to implement and use hospital@home care models? • Are healthcare providers sufficiently prepared to implement and apply hospital@home care models?

## 3. Results

### 3.1. Sample

**Figure 2:**
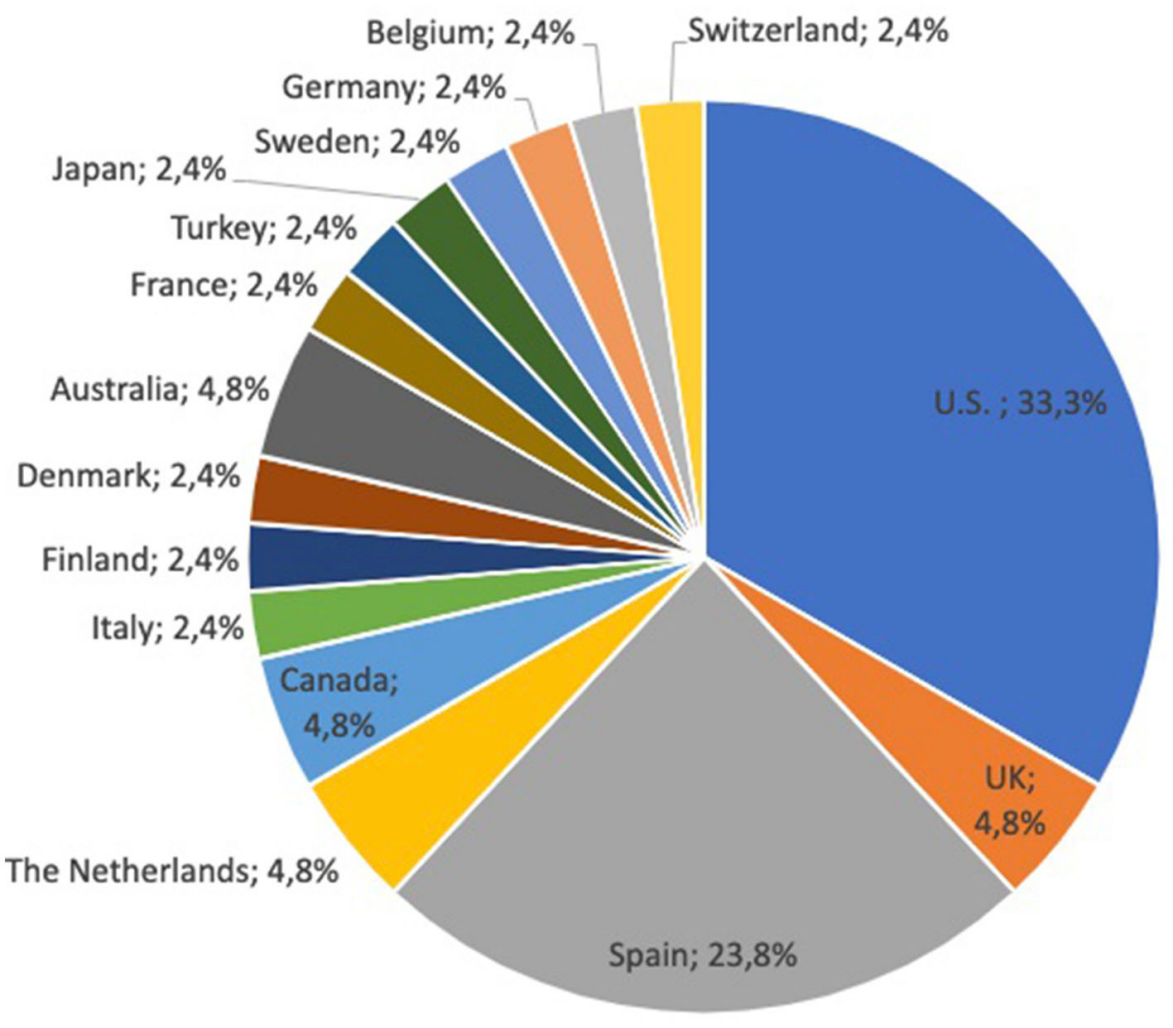
Countries for which hospital@home approaches were analyzed (*n* = 42).

The majority of articles introduce studies and concepts of hospital@home originated from the United States or Spain (see Figure 2). Multiple articles concerning the care concept were published by Mount Sinai Hospital in the United States. In total, articles from 16 different countries were included. Concerning the publication year, we can recognize that there is an increase in articles since 2017. Between 2013 and 2016, we considered only one or two articles per year, while between 2017 and 2022, an average of six articles were published per year.

In terms of practical orientation, 95.2% of articles (*n* = 40) reported on practical realizations of the care concepts, while only 4.8% of articles (*n* = 2) introduced concepts without a concrete practical realization. The settings of the hospital@home approaches comprise early discharge (31%, *n* = 13), treatment at home (59.5%, *n* = 25), and others (9.5%, *n* = 4). The latter include basically symptom monitoring approaches without a concrete treatment. A total of 23.8% of the articles mentioned aspects related to legal regulations which are payment models that were required or legal aspects that were relevant to run the hospital@home program.

### 3.2. Medical conditions

A total of 90% of the articles (*n* = 38) consider diseases related to adults, and 10% of the articles (*n* = 4) report on hospital@home implementations for children. A total of 20 different sets of medical conditions could be identified for which hospital@home realizations or concepts were reported or studied (see Table 2). A total of 28.6% of the articles did not specify the diseases they considered. Instead, they considered any acute medical condition. Treatment of patients with COVID-19 at home was reported in 11.9% of the articles, and treatment at home of patients with cancer was also reported in 11.9% of the articles. Hospital@home approaches for patients with hip fracture and orthogeriatric conditions as well as chronic obstructive pulmonary diseases were reported in two articles for each condition (4.8% per medical condition). The remaining 14 medical conditions occurred only once.

**Table 2:** Medical conditions for which hospital@home approaches were reported.

**Medical conditions**	**Number of approaches included**	**References to papers of the review**
Acute medical conditions	28.6%	(3, 10, 15–24)
COVID-19	11.9%	(25–29)
Cancer	11.9%	(30–34)
Hip fracture	4.8%	(35, 36)
COPD	4.8%	(37, 38)
Orthogeriatric conditions	4.8%	(39, 40)
Inflammatory or malignant bowel disease	2.4%	(41)
Hematological malignancies	2.4%	(42)
Neuromuscular disease with respiratory tract infections	2.4%	(43)
Congestive heart failure, chronic obstructive pulmonary disease, community acquired pneumonia, diabetic foot ulcer, complicated wound care	2.4%	(44)
Chronic pain and severe disabling spasticity	2.4%	(45)
Nosocomial infections	2.4%	(46)
Multiple sclerosis	2.4%	(47)
Pediatric care	2.4%	(48)
COPD, congestive heart failure, deep vein thrombosis, asthma, community-acquired pneumonia	2.4%	(49)
Heart failure, endocarditis	2.4%	(50)
Gastroenteritis	2.4%	(51)
Artificial heart-supported patients	2.4%	(52)
Schizophrenia, bipolar mood, unipolar mood, neurotic disorders	2.4%	(53)
Heart failure	2.4%	(54)

### 3.3. Stakeholder involved

Table 3 shows the stakeholder involved in delivering the care in the home. We can recognize that the core team of hospital@home formal caregivers were physicians and nurses. However, not all hospital@home care models involved at least these two care providers. There are some approaches that only rely upon informal caregivers monitoring the patient (e.g., spouses and children of the patient). Nurse practitioners and therapists were involved in around 26% of the care models; therapists were involved a bit less (21%). Informal caregivers were mentioned as part of the care team in 21% of the articles. Personnel for administrative and management tasks were employed in 19% of the care models. The involvement of pharmacists or the general practitioner (GP) was mentioned in only three articles (7.1%). Community paramedics were part of six hospital@home care models (i.e., 14.3%). Home care nurses and ambulatory care services were mentioned only once in the published research as providing care. In two articles, we were unable to characterize the staff involved.

**Table 3:** Stakeholder involved in the care of a patient.

**Stakeholder**	**Total number of approaches**	**Percentage (*n* = 42)**
Nurse	29	69.0%
Physician	29	69.0%
Therapists	11	26.2%
Nurse practitioners	11	26.2%
Social worker	9	21.4%
Management staff	8	19.0%
Informal caregiver	8	19.0%
Community paramedics	6	14.3%
General practitioner	3	7.1%
Pharmacist	3	7.1%
Insufficient information	2	4.8%
Emergency department	2	4.8%
Home care nurse	1	2.4%
Ambulatory care services	1	2.4%

Physicians include specialists; therapists include any kind of therapist (e.g., occupational therapist, physiotherapist, and dietitian).

### 3.4. Delivered services and first point of contact

Different services are provided by the various hospital@home programs. We group them into services related to diagnostics, monitoring, and treatment, accompanying services, and emergency handling. We list some example implementations per group as follows:

- Diagnostics-related services: point of care diagnostics [e.g., blood testing or tele-ultrasound (45)],
- Monitoring-related services: video or phone appointments with nurses or physicians (19), regular nurse or nurse practitioner visits (16) or community paramedicine visits (28), and telemonitoring of vital signs and parameters (10),
- Treatment-related services: administration of low-risk medications (30), palliative care support (29), administration of intravenous medicine including chemotherapy (16) or oxygen (10), rehabilitation either in person (15) or through a digital platform (35), and regular visits of therapists (41),
- Accompanying services: visits from social workers when needed (19) and patient education (41),
- Emergency handling: 24/7 telephone hotline for emergencies (18).

As the first point of contact, most hospital@home care models relied upon an emergency department of a hospital (15). One approach used a telephone triage (51) and other models discharged early from a hospital ward to the hospital@home (50).

### 3.5. Digital tools and technologies

In 20 articles (47.6%), we found information on the involvement of technologies to realize the hospital@home approach. Four of the articles reported on telecommunication tools that were used to communicate with the patient (e.g., by telephone and video communication systems). Integration with the electronic health record (EHR; e.g., for identification of potential candidates for early discharge to home) (54) or adaption of the EHR to document the treatment in the virtual ward were also reported. Cabrera López et al. (48) used audiovisual material and written documentation to support the education of patients and informal caregivers. Another approach used an online platform to deliver the intervention (rehabilitation exercises) (35). Three hospital@home models used a medication management system (31) or symptom monitoring system (33, 34). Few studies described hospital@home models that included wearables, biosensors, or more innovative digital tools, for example, a skin patch (VitalConnect) was used to monitor vital parameters along with machine learning to monitor health conditions (10), and electronic devices were used to measure vital signs (heart rate and blood pressure) (37, 49, 50).

### 3.6. Strengths, weaknesses, opportunities, and threats

Data about strengths, weaknesses, opportunities, and threats were extracted from the articles. They are summarized in the following sections.

#### 3.6.1. Strengths

Interestingly, many studies confirmed that the provision of care through hospital@home is equivalent to the one in hospital care in terms of mortality and/or rehospitalization. The clear strengths of hospital@home approaches are as follows:

- Hospital-related complications (delirium, falls, and nosocomial infections) (44) are reduced.
- The number of rehospitalizations and emergency department visits can be reduced compared to hospitalization (19).
- Hospital admission can be avoided or early assisted discharge (3) is possible using a hospital@home care model.

Several studies included in this review confirmed this. For the patient, additional benefits can be achieved. The care process can be better tailored to the patient's individual needs. There is closer contact between the patient, therapist, or nurse practitioner. This can be achieved through telemonitoring, which allows for earlier detection of potential complications arising from the patient's health condition (41). As long as the same care team is in contact with the patient, trust in the care team is higher and increases over time. One study described how patients appreciated visits by the same care team members over the course of the hospital@home caregiving period. Sending differing healthcare professionals each day was not well-accepted (32).

Some of the studies identified an increase in patient satisfaction and improvements in health-related quality of life when patients were admitted to the hospital@home service. Patients experienced increased autonomy and dignity when treated at home (17). Improved outcomes can be achieved due to increased physical activity of patients in the home (23) and through external factors contributing to wellbeing, e.g., pets, the presence of an informal caregiver, and being in a familiar environment. When continuous treatment is required, such as chemotherapy, unnecessary patient travel can be avoided, when this treatment can be provided at home. Treatment delays can be avoided, when rare resources at hospitals are replaced by corresponding equipment in the patient's home (e.g., a bed to sleep in) and when technology is involved and used to provide continuous symptom monitoring instead of scarce nurses. This can be the case in crisis situations such as during the COVID-19 pandemic. The hospital@home care model has the potential to prevent the collapse of the healthcare system in such situations (25).

For patients with infectious diseases, hospitals do not have to block beds to avoid transmission of infectious diseases when patients can stay in their personal homes and avoid contact with infected individuals. Here, hospital@home technologies could allow for remote monitoring of the development of a condition to avoid unnecessary use of health services or hospitalization (55). Few studies are available showing that hospital@home care is less expensive than hospital care (37). Thus, the optimization of limited resources in hospitals becomes possible through hospital@home care models (26). A cost-intensive hospital infrastructure can be reduced using hospital@home or the patient's own technologies could be integrated into a hospital@home model to reduce costs (56). This is the case as more and more individuals are buying consumer technologies to support aging in the home. Patients could use their own technology rather than the healthcare system buying technology for use so that societal costs are reduced (57).

#### 3.6.2. Weaknesses

A relevant weakness is the selection of patients suited for hospital@home care models. Clinical instability of a patient or the absence of adequate social conditions at home (46) prevents discharge to the home. Some hospital@home approaches highly depend on family members as caregivers. In this way, an increased burden is placed on family members involved in the process to a certain degree (43). Wait time for nurse visits or care team visits (32) might occur, impacting the daily routine at home or treatment of a health condition. When automatic tools generate alarms, the success of the approach depends on the appropriate reaction of the nurse practitioner or physician (i.e., the individual that is supposed to act upon the alarm) (34). However, without an appropriate reaction, patient harm can be caused. Approaches to hospital@home that rely upon a daily virtual nurse visit may miss critical changes in the patient's health status since the patient has to report their health status. In this way, there is a risk of a delay in necessary hospital admission (16). Challenges may arise in response to managing certain conditions, such as pain, which may arise as an issue if there is less frequent contact between nurses and patients (20). There is also a need to study human and technology fit (i.e., fit between the technology, the patient, and their caregiver preferences and supports in the home). Thus far, hospital@home care models seem to ignore patient involvement during the development phase of the care model. At least we could not identify a article reporting on patient involvement in development. The potential of participatory health informatics remained unconsidered even though the concept of hospital@home foresees comprehensive patient involvement.

#### 3.6.3. Opportunities

As healthcare staff shortages continue to worsen, there is a need for the optimization of processes without risking a drop in the quality of care. The studies that have been conducted around hospital@home demonstrate that it is possible to address this challenge and to shift treatment to personal homes as well as enable treatment at home or facilitate an early discharge from the hospital. An additional decrease in patient risks caused by hospital stays can also be achieved (12), for example, by reducing the risk of a hospital-associated patient fall. The COVID-19 pandemic has demonstrated that there is a great need for innovative and alternative care models to prevent the collapse of a healthcare system that is in crisis. COVID-19 has also shown us that telemedicine and telemonitoring activities can be used to alter the way in which we provide healthcare (55).

Many existing approaches to hospital@home have not fully explored the potential of telemonitoring using sensors. Including sensors in our constructions of care concepts and models could facilitate monitoring at home. Machine-learning algorithms have become more reliable for the automatic analysis of sensor data. Other opportunities to optimize the hospital@home care model and to scale it up for routine care are focused on the ongoing digitalization in healthcare and the availability of mobile health applications. There is research ongoing on digital health interventions that are made available through (mobile) health applications. We argue that these applications might well be applicable in the hospital@home settings (58). The hospital@home care models and their development can benefit from participatory health informatics by involving patients, caregivers, and healthcare professionals in the development of the care model and accompanying technologies. In this way, supporting technology can be developed which can empower patients to take a more active role in managing their health and wellbeing.

#### 3.6.4. Threats

There are several external features that may prevent further real-world implementations of hospital@home. These are related to cost models, acceptance by healthcare providers and patients, the development of virtual collaborations among care providers, and human as well as logistical challenges.

Acceptance of the new care model is necessary to become successful. This includes acceptance by health insurance organizations (private and/or public), by the care team (formal caregivers), by patient families (informal caregivers), and by patients. A reason for limited acceptance arises from concerns about the effectiveness of care at home (23). Another reason is that the hospital@home care model represents an intrusion into personal homes, i.e., into the intimate environment of an individual. Individuals might feel discomfort having strangers at home (23), for example, the home has to be cleaned up when physicians or nurses come home which produces stress in patients (32). In addition to this, the patient and/or family members may feel anxious about their ability to provide care in the home (59). Another serious patient concern could be data privacy of the patient, especially when mobile health applications are involved that process and transfer data *via* the Internet. Consequently, the transfer of the overall care model can be seriously impaired or even prevented from being implemented in the patient's home environment.

In addition to the care team and health insurance organizational acceptance of a new care model, there may be a need for financial incentives and demonstrated cost savings that would lead to the implementation of hospital@home on a larger scale (44). Other issues that may impact the implementation of hospital@home models include concerns surrounding patient safety and ensuring high-quality outcomes which involve determining the types of patients that would most benefit from this type of care. A careful selection of patients to be admitted to hospital@home reduces the occurrence of risks (26). To ensure the careful selection of patients in daily practice as well as corresponding decisions, models need to be more fully developed. This may include care teams conducting a home safety assessment (21) to ensure patient safety. To understand and follow instructions, patients would need an appropriate level of health literacy and adherence (21).

Implementing hospital@home approaches requires adequate education and knowledge of how and when to use digital tools. The tasks and responsibilities of healthcare professionals are different from those in the hospital care setting. Flexibility is required to deal with various situations. In contrast to a standard patient room in a hospital, home environments will be very different from each other; the available infrastructure might be different for each patient (31). Appropriate training of providers (nurses, general practitioners, and other involved staff) is required (16, 19, 60). When family members or other informal caregivers are supposed to take over the tasks of medical staff, they also have to be educated about health, healthcare, and digital tools.

The feasibility of clinical models of care provided in home-based settings has been shown in the studies, but they have to be carefully developed (26). A challenge is to scale the approaches up and integrate them into routine care (19). This requires the prediction of costs of the care itself, but also investments in equipment and education and training of care providers to enable them in a hospital@home context. In general, further work in the evaluation of the costs and benefits of hospital@home is needed. In addition, the involvement of technology like sensors and machine learning for analyzing sensor data and creating alerts requires technology acceptance by potential users (patients, nurses, and physicians). The success of such technologies depends on the level of digital literacy of caregivers and patients.

Many existing approaches rely upon the availability of a caregiver such as a family member at home (40). Adequate training of family members in therapeutic techniques is essential for ensuring patient safety (48). It must be noted that society is changing with more people living alone at home. Accordingly, the unavailability of a caregiver might become a threat to successful hospital@home implementations.

There is also a need for adapted clinical pathways and the transition to the primary care provider that needs to be considered if he/she is not involved in the hospital@home care model (21). In terms of documentation, appropriate information systems have to be integrated into the pathway. In case, the care model is a virtual ward of the hospital, an integration with the EHR maintained by the hospital is essential (33). For other care models, it is still an open issue as to how to document the treatment that takes place in the home (i.e., a home care record in terms of the types of data it collects would not be equivalent to an electronic health record used to document care in an acute care facility). We have seen that there are multiple stakeholders involved in a hospital@home setting. Communication among these stakeholders is essential for success (48): it requires close collaboration between all involved stakeholders and it involves resource allocation, economy, and cultural and legal issues (e.g., legal issues regarding responsibility for the patients in hospital@home care) (16, 60).

## 4. Discussion

### 4.1. Principal findings

The main finding of this review is that digital solutions and emerging concepts such as participatory health informatics are not yet considered much in hospital@home implementations, although they provide great opportunities for the future. Neither the involvement of patients in the development of hospital@home concepts was mentioned nor did we identify specific approaches (including technologies) that enable patients to become an active part of the treatment process within hospital@home. Monitoring of patients takes place basically through visits by healthcare professionals. No continuous monitoring is realized as mobile sensors have not been implemented to achieve this. Most approaches try to bring hospital care to the patient's home, missing opportunities that emerging technologies could deliver. The approaches often include a 24/7 phone hotline for emergency management. Only one approach was identified that used wearables in combination with machine learning for realizing continuous monitoring of vital signs (10). Surprisingly, another observation is that patients—treated at home, probably being alone in their home environment—are not even equipped with the supportive means to self-judge their health status in an objective manner. This is completely in contrast to the developments around mobile health where individuals are equipped with mobile applications and sensors to manage their diseases (58). Furthermore, the literature does not take into account the research that specifically focuses on digital health interventions to be used as a complement to hospital@home care concepts and care models. Only one article described a digital health intervention for rehabilitation exercises provided through a virtual platform (35).

### 4.2. Practical and research implications

Similar to our review, Spina et al. (59) already found out that to date none of the identified hospital@home literature reported on the involvement of patients and informal caregivers in the development, implementation, or evaluation of the care concept. As well, few studies have documented effective care models or human and digital resourcing approaches that have been successfully tested or implemented. Participatory design becomes more popular in health informatics research. It is grounded in ideas that consider a patient's needs and desires and this would help to create applications to which patients adhere (61). In addition, involving patients in the development and use of technology for hospital@home can help to empower patients to take an active role in their own care, and to manage their own health more effectively. This can be particularly important for patients who are managing chronic conditions, as they may require ongoing care and support, and may benefit from being able to access and use health information technology in their own homes.

However, our results show that this trend has not yet emerged in the hospital@home literature. Future research on hospital@home should consider patient and caregiver participation, including tools and methods used in participatory health informatics. There are different perspectives, concerns, and viewpoints related to possible implementations of hospital@home. A participatory design approach can help to consider such viewpoints and consider them in concept development and realization (62). It can also contribute to acceptance. Acceptance has been identified as a threat to successful hospital@home care models. Involving all stakeholders in the development of hospital@home concepts might contribute to acceptance. Of course, financial models are also of relevance for acceptance by healthcare professionals.

Patient safety is essential in healthcare and equally in hospital@home settings. Research is required on the careful selection of patients who are treated at home. This selection should not only consider clinical parameters, but also social and cognitive aspects. Only limited information was provided on the patient selection process within hospital@home care models which is probably due to the clinical trial settings in the assessed studies where eligibility criteria are specified. More studies are needed for identifying medical conditions suited to be treated at home. Currently, most research is available on acute medical conditions. Evidence for other medical conditions is still missing. In addition to the medical condition and the patient's health status, aspects such as health literacy and (when technology is used) the eHealth literacy of the patients have to be assessed to judge whether a treatment-compliant behavior can be expected. The assessment also has to consider the home environment. Only one article included in this review mentioned explicitly a home safety assessment (21). For daily practice, standard operating procedures would help in doing the patient assessment in a standardized manner and in this way ensure patient safety. Another option would be the automation of patient selection (12).

Education is another aspect to be considered before hospital@home care models are released in daily practice. Patients at home need to learn how to react appropriately in certain situations and how to monitor their health. Hospital@home requires adequate informal support and patient and caregiver education because it involves providing care to patients in their own homes rather than in a traditional hospital setting. Patients and their carers may need to manage their treatment more actively by handling things like drug administration, symptom monitoring, and communication with healthcare professionals. Inadequate education can lead to poor outcomes and can also lead to patients and caregivers feeling overwhelmed and stressed by their care responsibilities, which can negatively impact their overall wellbeing and impact on the treatment outcome. This can disproportionately affect certain populations, such as low-income individuals, elderly individuals, and individuals with limited literacy or language skills. Therefore, it is important for hospital@home programs to ensure that adequate informal support and patient/caregiver education are available and accessible to all patients and caregivers. Technology, for example, in terms of a conversational agent could be used to answer patient questions even when they are at home. Additionally, healthcare professionals need to be educated in new care models. Especially when technology is involved, interacting with the technology must be learned as well as analyzing and interpreting results provided by the technologies.

Another huge research topic is the involvement of technology in hospital@home care models. The use of technology for remote patient monitoring has already been considered an opportunity for hospital@home by other researchers (12). Predictive algorithms based on vital signs and activity levels can be used for monitoring or prediction of health events. There is also a need for hospital@home concepts that allow for equitable access to care (12). Some of the approaches explicitly required that patients live within a certain distance of the hospital. Such models are not applicable to rural areas. However, there are still several challenges to be overcome before technologies can be well-integrated into hospital@home with attention to health equity. To be able to create new innovations in the context of hospital@home, the development process should be agile. An agile process allows one to react quickly to new technological achievements and changing requirements (63). For example, new sensors could be released to market that would be useful to be integrated into a hospital@home solution under development.

Integration of the individual digital ecosystem with the institutional digital health ecosystem would be beneficial (56). Thus far, the healthcare technology infrastructures are still siloed and disconnected. We recognize a huge potential to develop hospital@home further in this direction to make use of the opportunities. Leff et al. (11) also suggest technology use and telehealth in their research agenda for hospital@home care models. They suggest there is a need to study barriers to technology use, to define standards and consider cybersecurity. Technology has to be integrated optimally into the hospital@home workflow. Without integration healthcare professionals, patients and caregivers will miss out on potential uses of technologies. The patient should be equipped with the means to be an active part of treatment and be able to easily use the technologies used to collect or manage their healthcare data (64). We can imagine an approach that equips the patient with a mobile health app that allows monitoring sensor data, which are visualized in an appropriate manner. Together with the personal perception of health at a specific point in time, a patient can much better judge whether help is needed, and whether an emergency hotline has to be called. This holds, in particular, true when no informal caregiver is available (but even the caregiver could benefit from a mobile health app). There is a need to assess whether a complete automatic analysis of sensor data through machine learning algorithms would be accepted by patients. A risk of such an automatic approach is that the patient is no longer involved in self-assessing his or her body and health but can rely upon the data from the sensors.

Integrating monitoring technologies could allow for continuous monitoring instead of a nurse visit once a day which would contribute to safety. A quick reaction to worsening situations of the current health status highly depends on a patient or informal caregiver that calls for help.

There are several moral, social, and ethical aspects that have to be considered in the context of hospital@home (11) but they were not considered in the assessed studies. A reason again might be the development and testing in a lab setting followed by clinical trials and then naturalistic studies (64). Before such models will be implemented at a regional and national level, these aspects have to be considered. In all included studies, patients had to give their consent to being treated at home. This raises the question of whether there will be enough time in daily practice to answer all the questions of patients before they give their informed consent for home treatment. We argue that corresponding protocols have to be implemented to ensure appropriate information about patients before decision-making.

In summary, future developments around hospital@home should consider the following aspects:

- Follow a participatory design approach, i.e., involve patients and all other relevant stakeholders in the design, development, and testing of solutions.
- Identify medical conditions and create guidelines on how to select patients to be included in hospital@home programs.
- Follow an agile development and design process to make sure that the latest developments can be considered and test technologies to support monitoring and care at home.
- Develop education programs for patients and their caregivers.
- Develop education programs for other stakeholders (physicians, nurses, and the like).

### 4.3. Strengths and limitations of this study

This is, to our knowledge, the first review article specifically studying technology use in hospital@home implementations as well as studying strengths, weaknesses, opportunities, and threats of hospital@home. In this way, this study offers concrete starting points for future research in this field as well as useful implications for practitioners. The overall study relies upon a systematic, comprehensive, and comparable literature search as well as a SWOT analysis showing relevant aspects to be considered. However, our study does not come without limitations.

The search of this review was restricted to articles in English. There were several articles that might have been relevant but provided only an English abstract and full text in another language, such as Italian or Spanish. Accordingly, it is likely that we missed relevant publications. We did not have two reviewers independently assess citations for inclusion which would have reduced the risk of biased inclusion of articles. Moreover, some articles were referring to the same hospital@home approach (e.g., the Mount Sinai hospital program). When calculating percentages, we did not merge these articles referring to the same care model. This slightly impacts the reported percentages. We resisted comparing the care models across countries. In particular, the involved healthcare professionals might differ depending on the national healthcare system. For example, in Switzerland, the concept of a family doctor is well-established and even supported by health insurance models that offer reduced fees when assigned the insurance model in favor of a family doctor as the first point of contact.

Articles on hospital@home approaches related to palliative care or midwifery were explicitly excluded. We argue that the care required and provided in these contexts is very specific, which was the reason for exclusion. In addition, we only included one literature database, namely PubMed. This could also let us miss relevant publications and thus impair the external validity of our results. If in future specific technical solutions to hospital@home are of interest, the consideration of literature databases such as IEEE Xplore or ACM Digital Library is highly recommended.

## 5. Conclusion

In this study, we analyzed the current landscape of hospital@home implementations with a special focus on the use of technology and consideration of participatory approaches. Hospital@home has already been implemented in practice for several years at least in some countries like the United States and Spain. However, participatory health informatics and technology use is still rare if integrated at all. Thus, we argue that the integration of digital health in hospital@home care models is still a vision. Thus far, the potential of technologies and mobile health has not at all been used. Nevertheless, some attempts are starting, but there is still a lot of research to be done. Considering these emerging trends could help in addressing the threats we identified as part of this work. For instance, data transfer and data availability could contribute to good communication among healthcare professionals involved in the hospital@home treatment of a patient. We conclude with our recommendation that future studies and new concepts for hospital@home should consider technology use including mobile health and participatory health informatics.

## Author contributions

KD and RM planned the study, decided on the search strategy, and judged the articles regarding eligibility. KD conducted the search and performed data extraction. EB, AK, and KD wrote the discussion and the section on the SWOT analysis results. All authors critically revised the manuscript for important intellectual content, approved the manuscript for publication, and agreed to be accountable for all aspects of the work.
